# Integration of Lung Point-of-care Ultrasound into Clinical Decision Making for Medical Students in Simulated Cases

**DOI:** 10.5811/westjem.2020.12.48717

**Published:** 2020-12-14

**Authors:** Michelle Lum, Lauren Sheehy, Jason Lai, David Tillman, Sara Damewood, Jessica Schmidt

**Affiliations:** * University of Wisconsin, Department of Emergency Medicine, Madison, Wisconsin; † Tucson Medical Center, Department of Emergency Medicine, Tucson, Arizona

## Abstract

**Background:**

Point-of-care ultrasound (POCUS) has an emerging presence in medical student education; however, there is limited evidence that this translates into appropriate clinical care. We aimed to evaluate the ability of medical students to integrate newly obtained POCUS knowledge into simulated clinical cases.

**Methods:**

We conducted an observational study of medical students participating in a mandatory rotation during their clinical years. Students in small groups underwent formalized lung POCUS lectures and hands-on training. Students participated in simulated “dyspnea” cases focused on either congestive heart failure (CHF) or chronic obstructive pulmonary disease (COPD). They were observed for critical actions including elements related to medical decision-making and ultrasound use and interpretation. Ultrasound-specific written knowledge was gauged with a short assessment after the first lecture and at week 4.

**Results:**

A total of 62 students participated and were observed during simulations. All groups correctly identified and treated CHF in the simulated case. Most groups (7 out of 9) attempted to use ultrasound in the CHF case; five groups correctly recognized B-lines; and four groups correctly interpreted B-lines as pulmonary edema. No groups used ultrasound in the COPD case.

**Conclusion:**

Most students attempted to use ultrasound during simulated CHF cases after a brief didactic intervention; however, many students struggled with clinical application. Interestingly, no students recognized the need to apply ultrasound for diagnosis and management of COPD. Future studies are needed to better understand how to optimize teaching for medical students to improve translation into POCUS skills and improved clinical practice.

## INTRODUCTION

Point-of-care ultrasound (POCUS) is an important diagnostic tool that has been adopted by physicians in several clinical specialties.^1^ POCUS describes the use of ultrasound in a focused and goal-directed manner for a specific clinical context but does not encompass an entire comprehensive radiologic evaluation.^2^ Given its clinical utility, calls have been made to incorporate POCUS education into medical school curricula, and a growing number of medical schools have done just that.^3^–^5^ As Solomon et al wrote in the *New England Journal of Medicine*, “A generation of physicians will need to be trained to view this technology as an extension of their senses, just as many generations have viewed the stethoscope.”^6^ The future state of POCUS depends on training at all levels of medical education.

Several studies have shown that medical students are able learners and can be taught to perform POCUS with short didactic training.^7^–^12^ Yet there has been limited investigation of how POCUS teaching translates to clinical decision-making and practice. Several authors have emphasized that new technology does not always correlate favorably with clinical care.^13^,^14^ Understanding how medical students carry novel skills with them into clinical practice is essential to appropriately developing training curricula.^15^ The goal of teaching is to have students progress along Miller’s framework from knowledge, to competence, to performance.^16^ High-fidelity simulation offers a venue to view learners’ performance of POCUS. It also has the advantage over other assessment techniques of evaluating students in a controlled clinical environment.^17^–^22^ With regard to POCUS, simulated case scenarios provide an ideal method of observing skill translation in a controlled study setting.

We aimed to evaluate the ability of medical students in their clinical years to integrate newly obtained knowledge related to lung POCUS into clinical decision-making. While it has already been shown that students can readily acquire POCUS knowledge, we hypothesized that students may struggle to integrate POCUS into simulated case scenarios. We aimed to evaluate appropriate use of POCUS by observing students’ ability to correctly acquire and interpret images and make appropriate changes in clinical management based on their findings.

## METHODS

This study was an observational study of medical students at the University of Wisconsin, a large medical school in the Midwest. The study was conducted from January 2019–January 2020. At our institution, all medical students participate in a 12-week mandatory rotation termed the Acute Care Block (ACB), which includes multispecialty education in emergency medicine (EM), neurology, internal medicine, radiology, and psychiatry. The study was determined to be exempt by the University of Wisconsin Institutional Review Board and was approved by members of the medical school ACB curriculum team.

### Curriculum Design

#### Lectures

We designed a lecture and hands-on training focused on lung POCUS targeted toward novice users. The lecture covered ultrasound physics, imaging modalities, artifacts, probe selection, and basic lung ultrasound including evaluation of lung sliding, A-lines, B-lines, and clinical correlates. Lectures also emphasized the use of lung POCUS in an undifferentiated dyspneic patient, including evaluation of pathologies such as pneumothorax and alveolar interstitial syndrome. Lectures were taught by faculty from radiology and EM. Students then practiced hands-on scanning skills with bedside instructors on live standardized patients (who were without pathology), and key concepts were reviewed. Students previously received core content instruction on the pathophysiology, clinical manifestations, and management of congestive heart failure (CHF) and chronic obstructive pulmonary disease (COPD) as part of the standard medical school curriculum. This content was designed and reviewed by a group of faculty physician educators within internal medicine, EM, and radiology.

**Population Health Research Capsule**

What do we already know about this issue?

We know that medical students are able learners of point-of-care ultrasound (POCUS) with only brief educational interventions.

What was the research question?

Will medical students successfully integrate newly obtained lung POCUS knowledge into medical decision making?

What was the major finding of the study?

Students struggled to translate knowledge of lung POCUS into performance in a simulated clinical scenario.

How does this improve population health?

Evaluating students’ knowledge translation of POCUS skills allows us to improve targeted medical student didactics and refocus POCUS education on patient outcomes.

#### Dyspnea simulation

Students worked in groups of 3–5 students. Each group participated in one of two high-fidelity simulation cases relating to a patient with dyspnea. Cases were developed by a multidisciplinary educational faculty team specifically for the ACB rotation. In both cases, patients presented with a chief complaint of “shortness of breath.” In the first case, CHF, the patient had heart failure and presented with physical exam findings consistent with fluid overload including pitting lower extremity edema, bibasilar crackles, and low oxygen saturation. In the second case, the patient had COPD and presented with physical exam findings consistent with exacerbation, including wheezing. Students were not aware of a final diagnosis or pathologic findings prior to the case, and were only given the simulated patient’s age, gender, and chief complaint of “shortness of breath” immediately prior to the start of the simulation.

#### Simulated POCUS

For both CHF and COPD simulation cases, an ultrasound machine was placed in the simulation room. If students chose to use the ultrasound, they were prompted by the simulation facilitator to describe where they were placing the probe and what structures or findings they were expecting to visualize. We incorporated a slight delay in obtaining a chest radiograph (CXR) during the simulation to allow the students the opportunity to incorporate POCUS. Because the simulation mannequin did not produce ultrasound images, static images were presented to students on an in-room display screen in the same way that CXR, laboratory studies, or other diagnostic data were presented. Static images were used in lieu of video clips due to technical limitations of the available equipment.

### Assessment

#### Knowledge-based

Students were administered a short, knowledge-based assessment immediately after the first didactic session on week 1 to evaluate POCUS knowledge acquisition. This assessment included questions specific to lung ultrasound interpretation and clinical management based on POCUS images. Students were administered an identical assessment, after the simulation exercise, in week 4 to assess for knowledge retention. Survey collection was performed using SurveyMonkey (San Mateo, CA). The assessment was created and reviewed by two ultrasound fellowship-trained faculty members.

#### Simulation

Simulations occurred during week 4 and were video recorded. Coding was completed using a predetermined rubric (Appendix A) detailing potential actions by two independent reviewers (LS and ML). This rubric was adapted from previously described checklists related to simulation and modified to our learners’ training level.^23^,^24^ Discrepancies were mitigated by a third reviewer (JS). Reviewers noted whether students completed a specific task (eg, listen for breath sounds), and time of occurrence. If students performed a task more than once, only the first occurrence was documented. When students discussed but did not initiate a task (eg, initiating bilevel positive airway pressure), time was noted for first discussion and then, if applicable, for completed action.

#### Ultrasound-specific scoring

To assess the use of POCUS in a clinical scenario, the scoring rubric included tasks specific to ultrasound adapted from the Emergency Ultrasound Standard Reporting Guidelines: ACEP 2018.^25^ These included correct probe selection, appropriate probe position, appropriate probe orientation, verbalized recognition of B-lines on images provided, and verbalized recognition of pulmonary edema.

### Analysis

We compiled knowledge assessments in Microsoft Excel (Microsoft Corp, Redmond, WA) and performed statistical analyses using a paired two-sample for means t-test with a 95% confidence interval and interquartile ranges.

## RESULTS

A total of 62 students participated in the ACB course during the study period. All students agreed to and were observed and evaluated during clinical simulation. Week 1 and week 4 knowledge assessment data was available for 50 individual students from July 2019–January 2020. We did not include 12 students’ data due to missing data (overall response rate 81%). Reasons for missing data include technical difficulties.

### Brief Knowledge-assessment Results

Overall, most students were able to correctly identify normal lung images, including lung sliding (70% week 1 and 74% on week 4, *P* = 0.62) and could identify correct medical management (use of diuretics) in a patient with B-lines on ultrasound (86% week 1 and 82% week 4, *P* = 0.60). Students did demonstrate some knowledge decay from week 1 to week4 with regard to recognition of specific lung pathologies, including pneumothorax with M-mode (88% on week 1 and 70% on week 4, *P* = 0.038).

### Simulation Results

Students in 18 unique groups of 3–5 students participated in dyspnea simulation cases over the course of the study. Half (nine) of these groups participated in a COPD case simulation, and the other nine of these groups participated in a CHF case simulation. A total of 32 students participated in the COPD cases, and a total of 30 students participated in the CHF cases. During the simulated COPD cases, none of the groups used ultrasound. During the nine CHF case simulations, 89% of groups (8) discussed use of POCUS, and 78% (7) attempted to use the machine provided in the simulation room. Four groups (44%) chose the correct curvilinear probe to assess for B-lines. All groups that attempted ultrasound applied the probe to the anterior chest, although two groups were attempting echocardiography, for which they had not been formally trained in this course. We found that 55% of groups (5) were able to correctly identify B-lines. Three of these groups correctly verbalized that B-lines represent pulmonary edema. One group did not articulate the identification of pulmonary edema until it was confirmed on CXR. Another group incorrectly identified B-lines as representative of pneumothorax. Two groups did not use POCUS, but both of these groups correctly diagnosed CHF clinically prior to obtaining laboratory or imaging results. Most groups (6 out of 9) administered diuretics before any imaging resulted. The other three groups obtained POCUS images prior to diuretic administration. All groups correctly identified the diagnosis of CHF and treated appropriately with diuretics. In the COPD cases, 88% (8) of the groups initiated nebulizer treatments prior to any imaging being performed. Table 1 describes the average time for groups to perform critical actions during the simulated CHF case. Table 2 describes the number of groups who successfully completed ultrasound-specific actions. Timing of ultrasound is not shown for COPD cases as no groups attempted ultrasound.

## DISCUSSION

As POCUS education continues to expand in medical schools, understanding how medical students will incorporate POCUS into their clinical decision-making is increasingly important. Some authors have raised concerns that acquiring information in lecture format may not actually translate into improved clinical skills. Feilchenfeld et al have previously commented: “the rationale that ultrasound training in [undergraduate medical education] will improve the quality of patient care was difficult to evaluate.”^14^

Our study reflected previous findings that students readily gained POCUS knowledge after a brief didactic session. Overall, students were able to identify normal lung anatomy with lung sliding (74%) and lung pathology with B-lines (82%); however, they were inconsistent during simulation with correct probe selection (only 44% correct) and recognition of B-lines (56%). This may have been due to confusion between linear probe selection for evaluation of pneumothorax and curvilinear probe selection for B-lines, or our limitation of static images used during simulation due to technical issues. Differences in written knowledge assessment and simulations performance may also be due to increased cognitive burden between the two assessment modalities as further discussed below.

We also found that students demonstrated inconsistent application of POCUS in simulation. While most students did discuss using POCUS during the simulated CHF case (8 of 9), only seven of the groups went on to actually attempt image acquisition. Interestingly, although the students were trained specifically on lung POCUS, two of the groups attempted cardiac ultrasound instead of lung. This likely reflects additional knowledge students acquired during clinical rotations and observations of clinical practice. None of the groups attempted to use POCUS for the case of COPD. This is surprising as evaluating for pneumothorax or a cardiac etiology of wheeze is still important in an undifferentiated dyspneic patient. Students’ decision not to use POCUS here may reflect their inexperience in approaching an undifferentiated patient. Alternatively, it may reflect a lower comfort level with POCUS—having had a few weeks pass since their last POCUS teaching—and an over-reliance on skills they were more comfortable with, such as physical exam.

Another interesting finding was that most groups provided medical management before any imaging was obtained—ultrasound or CXR. This could reflect the students’ inexperience with creating differential diagnoses, or conversely that the cases were relatively straightforward and students recognized the need for correct clinical management based primarily on physical exam and vital signs.

Medical students early in their clinical training, such as our group, have limited in vivo clinical experiences to draw from; it may have been difficult for students to approach the simulations as they would have a live clinical scenario, simply because they had encountered so few such scenarios in real clinical practice. Thus, the students’ decision not to use ultrasound in the COPD case and to generally initiate management prior to diagnostics in both the CHF and COPD cases may reflect a view of the simulation as a hidden list of tick boxes—tasks to be completed—rather than a real clinical scenario, requiring movement through a differential diagnosis.

Another possibility is that there was a mismatch between the cognitive complexity of the simulation and the learner. This has previously been discussed as a potential pitfall of simulation.^26^ Cognitive load theory would suggest that if learners have limited proficiency in the many tasks encountered in a simulation (eg, interpretation of vitals, interpretation of CXR, use of POCUS), then they will have difficulty translating any one of these skills into a clinically sound action. It is possible that in a lower complexity simulation, learners would have integrated ultrasound more robustly.

Overall, students in our study demonstrated difficulty translating *knowledge* of ultrasound into *performance* of ultrasound in a simulated clinical scenario. A number of possible explanations for this difficulty exist, including a lack of comfort with POCUS (due to insufficient hands-on training or training being too remote) and difficulty approaching the simulation as a real clinical scenario. It is also possible that the simulation was poorly matched to the learner—in terms of complexity—making a subtle transfer of POCUS skill more difficult to assess.

## LIMITATIONS

Our study did have several limitations. First, as a single-center, observational study, the ability to generalize these findings may be limited. Second, the curriculum may have been too limited to adequately prepare students for simulated clinical applications. Third, students were assessed via a simulated case scenario (with potential pitfalls as detailed above); this may not reflect how they would manage patients in real life. However, given the innumerable external variables involved with direct observation, we felt that high-fidelity simulation provided a reasonable approximation, with the benefit of environment control as previously mentioned. Students also completed simulated cases in groups, making it difficult to compare execution in this arena with scores on the knowledge assessment, which was completed individually. Finally, students were assessed early in their clinical training and may not have been adequately prepared to work through a differential diagnosis in real time.

## CONCLUSION

As medical schools continue to develop POCUS curricula for students, a greater understanding is needed as to how students will incorporate these skills into clinical decision-making. Our study demonstrated that students were able to acquire POCUS-related knowledge after a brief didactic session but struggled to apply this knowledge in simulated clinical scenarios. More research is needed to determine how best to move learners from POCUS knowledge acquisition to application, and to determine how best to assess this process.

## Supplementary Information



## Figures and Tables

**Table 1:** Timing of critical actions completed by student groups during congestive heart failure and chronic obstructive pulmonary disease simulated cases.

	CHF			COPD		

Patient care task	Completed by groups	Time (range)	Time (average)	Completed by groups	Time (range)	Time (average)
Request vital signs	100%	0:00–0:48	0:18	100%	0:20–1:31	0.40
Listen for breath sounds	100%	0:26–4:04	1:40	100%	0:27–2:48	1:22
Apply O_2_	100%	0:16–1:54	1:05	100%	0:23–2:05	1:11
Order diuretic	100%	5:27–16:57	6:41	0%	NA	NA
Order nebulizer	0%	NA	NA	100%	3:20–5:40	4:14
Request CXR	100%	3:02–11:00	9:36	100%	2:09–8:48	5:47
Use ultrasound	78%	6:51–13:51	9:23	0%	NA	NA

*CHF*, congestive heart failure; *COPD*, chronic obstructive pulmonary disease; *CXR*, chest radiograph.

**Table 2:** Proportion of groups completing ultrasound-specific actions.

Ultrasound skill	Completed by groups
Appropriate probe selection	44%
Appropriate probe position	78%
Recognize B-lines	56%
Recognize pulmonary edema	44%
