# Supplementary material for: Integration of Lung Point-of-care Ultrasound into Clinical Decision Making for Medical Students in Simulated Cases
Source: West J Emerg Med. 2020 Dec 14;22(1):124–9. doi: 10.5811/westjem.2020.12.48717 (PMC7806326; doi:10.5811/westjem.2020.12.48717)
Supplement: Supplementary file 1 [file wjem-22-124-s001.docx]

**Appendix A:** ACB Ultrasound Simulation: CHF Case

| **CLINICAL ASSESSMENT** | **TIME** |
| --- | --- |
| Case Start | 0:00 |
| Ask for vital signs |  |
| Listen for breath sounds |  |
| Recognize respiratory distress? (verbalize) |  |
| Apply supplemental oxygen |  |
| Request CXR |  |
| Request CT |  |
| Request/use ultrasound machine |  |
| Interpret ultrasound |  |
| Interpret CXR |  |
| Request Chest tube |  |
| Antibiotics given |  |
| Lasix given |  |
| Other treatment given (specify) |  |
| BiPAP |  |
| Intubation |  |
| Consultant - radiology |  |
| Consultant- pharmacy |  |
| Consultant- hospitalist/TLC |  |
| Consultant- other (specify) |  |
| Disposition (verbalize) |  |
| CPR |  |
| Other tests (specify) |  |
| Other interventions (specify) |  |

| **ULTRASOUND USAGE** | **Yes/ No** |
| --- | --- |
| Correct probe selection |  |
| Use of gel |  |
| Appropriate probe position |  |
| Appropriate probe orientation |  |
| Recognize B-lines (verbalize) |  |
| Recognize pulmonary edema (verbalize) |  |
